# Evaluation of a healthcare walk-in centre in an immigrant-dense area from the perspective of Swedish-born patients

**DOI:** 10.1017/S1463423621000189

**Published:** 2021-04-22

**Authors:** Rikard Wärdig, Emina Hadziabdic, Katarina Hjelm

**Affiliations:** 1 Department of Health, Medicine and Caring Sciences, Division of Nursing and Reproductive Health, Linköping University, Linköping, Sweden; 2 Department of Health and Caring Sciences, Linnaeus University, Växjö, Sweden; 3 Department of Public Health and Caring Sciences, Uppsala University, Uppsala, Sweden

**Keywords:** healthcare centre, primary health care, qualitative research, walk-in centre

## Abstract

**Aim:**

This study evaluates a healthcare walk-in centre in an immigrant-dense area from the perspective of Swedish-born patients.

**Background:**

The studied healthcare centre started a walk-in centre to increase healthcare accessibility for immigrants. This form of care is not primarily for Swedish-born patients although everyone is welcome. For this reason, it is important to evaluate the walk-in centre from different perspectives: the healthcare workers, the immigrant patients, and in this study focusing on the Swedish-born patients.

**Method:**

This qualitative exploratory study used content analysis to analyse data collected from semi-structured interviews. Semi-structured interviews were held with 12 purposively sampled Swedish-born patients visiting a healthcare centre in Sweden.

**Findings:**

Most informants characterised the care they received as professional and timely and noted that accessibility was the main reason they sought care at the walk-in centre. In addition, they noted that being able to seek care on the day they want creates a feeling of security. However, Swedish-born informants seemed to prefer a traditional healthcare centre, although they viewed the walk-in centre as legitimate because everyone has access to it.

**Conclusion:**

As the walk-in centre was perceived as having good accessibility, participants experienced that they could easily receive help for minor health problems. However, they also identified several ways the walk-in centre could be improved. For example, some participants preferred to remain outside while awaiting their turn to see a healthcare provider and wanted immigrant patients to leave their relatives at home when possible to minimise the risk of spreading infection. In addition, some participants thought a triage system could be implemented so that more severe cases could advance more quickly in the queue. The homogeneous sample of informants raises questions about whether this healthcare model is indeed accessible to everyone.

## Introduction

The Swedish Health and Medical Services Act states that primary healthcare (PHC) agencies, irrespective of illnesses, age, or patient groups, are responsible for providing basic medical treatment, nursing, prevention work, and rehabilitation that do not require a hospital’s medical and technical resources or other special skills (Ministry of Health and Social Affairs, 2017). The county councils have great freedom to decide how PHC should be organised since the act does not define the work of the PHC team (Swedish National Board of Health and Welfare, 2016).

The Swedish healthcare system is divided into three levels: the primary level with PHC; the county level with county hospitals and district county hospitals; and the regional level with regional/university hospitals. PHC is responsible for public health and treatment of diseases and injuries that do not require hospital or specialist care. PHC is provided in health centres staffed by GPs, nurses, and assistant nurses. Each PHC area serves a geographically defined population. Patients requiring specialised care are referred to hospitals, and the most highly specialised care is provided by university hospitals (Swedish National Board of Health and Welfare, 2016). The PHC tasks may differ at the level of detail between the different county councils. In the Swedish PHC system, healthcare centres are central as they conduct reception activities for planned and unplanned health care within general medical competence (Swedish National Board of Health and Welfare, 2016).

Access to PHC can be difficult for patients who are not fluent in Swedish, as much of the system is based on telephone conversations where patients must explain their symptoms before a healthcare provider can offer treatment options. To increase accessibility for immigrants who do not speak Swedish, the studied healthcare centre started a walk-in centre. The purpose of the walk-in centre was to give the immigrant population the same opportunities to access PHC as the native population (Swedish-born). In other words, there are immigrants as well as Swedish-born patients who visit the studied walk-in centre. Thus, it is important to evaluate the walk-in centre from different perspectives – the perspective of Swedish-born patients, the perspective of healthcare providers, and the perspective of immigrants. This investigation will focus on Swedish-born persons and is one in a series of studies. This study investigates what methods are suitable for meeting the needs of the Swedish-born patients and provides suggestions for how PHC can be organised.

## Background

Although PHC systems work differently around the world, systems based on PHC are effective in reducing disease and mortality and promote a more equitable distribution of health care worldwide (Starfield *et al.*, 2005). For example, time is one way to highlight these differences. A large proportion of the global population meet only a few minutes with their PHC physicians, ranging from 48 s in Bangladesh to 22 min in Sweden (Irving *et al.*, 2017).

PHC in the industrialised part of the world faces challenges from several directions. An ageing population means more people will suffer from more forms of chronic diseases (Nolte & McKee, 2008). At the same time, advances in medical science make it possible for many people to live longer despite multiple serious chronic health conditions (Osborn *et al.*, 2015). Added to this is the ongoing global migration, which is making many European societies multicultural (International Organization of Migration, 2018). The Swedish immigrant population is heterogeneous with over 200 different nationalities represented (Swedish Migration Agency, 2016). The immigrant population often experiences poorer health (Hemminki, 2014), and problems in accessing PHC (Cheng, Drillich & Schattner, 2015). These challenges facing PHC lead to a high-risk area for diagnostic errors (Singh *et al.*, 2017), which can lead to patient harm as a result of wrong or delayed treatment (Panesar *et al.*, 2016). In addition, a shortage of doctors means that nurses take on more responsibilities. Fortunately, care delivered by nurses in PHC has been shown to generate similar or better health outcomes compared to care delivered by doctors in a wide range of patient conditions, although nurses seem to spend more time with patients during consultations (Laurant *et al.*, 2018).

Walk-in centres have been evaluated to a limited extent. Some studies have evaluated nurse-led walk-in centres (Desborough, Forrest & Parker, 2011; Mannie, 2014) and others examine centres led by general practitioners (Jones, 2000; Salisbury & Monro 2003). Therefore, it is problematic to compare because the literature presents different contexts with different staff and different levels of care.Two review studies that include walk-in centres staffed by general practitioners or family physicians have been published (Jones, 2000; Salisbury & Monro, 2003), and is closest to the context that our study describes, in other words, that nurses carry out the primary assessments, and that doctors are available when needed. However, these results are not entirely comparable to the Swedish context since these walk-in centres are independent of a healthcare centre. As in Sweden, these walk-in centres were established to increase the accessibility of health care. Salisbury and Monro (2003) found that many types of people use walk-in centres. Often, these patients are seeking help for minor injuries and ailments. However, it is unclear whether increased accessibility is motivated by the real needs of patients or whether increased accessibility motivates people to seek the care they do not really need. Whether walk-in centres are nurse-led or GP-led, patient satisfaction seems high (Salisbury & Monro, 2003; Desborough, Forrest & Parker, 2011). An earlier Swedish study, evaluating a walk-in centre from the healthcare worker’s point of view showed that a walk-in centre can be seen as related to ensuring patient safety and delivering equal care for all. At the same time, it cannot be the only form of care offered, as it seems not to be adapted to certain groups, such as people with disabilities and the elderly (Wärdig *et al.*, 2019).

The main purpose of this series of studies is to evaluate whether a walk-in centre meets the needs of patients or whether this sort of care can be provided using more suitable measures.

## Aim

This study evaluates a healthcare walk-in centre in an immigrant-dense area from the perspective of Swedish-born patients.

## Methods

### Design

This qualitative exploratory study uses semi-structured interviews to collect data. This study provides new insights into a phenomenon where knowledge is limited (Patton, 2015). Semi-structured interviews were used as this format encourages participants to express their own experiences but within a given framework.

### Settings

The walk-in centre is physically located in the same building as the healthcare centre and belongs to the same organisation. The healthcare centre is in a medium-sized city in the southeast of Sweden and has approximately 18 800 patients listed. The district belongs to an area with a high proportion of immigrants. They represent many different countries of origin, (more than 200), and especially many have arrived from Syria, Afghanistan, and North Africa in recent years (Swedish Migration Agency, 2016). The walk-in centre was started because of the high number of foreign-born patients, who often are not fluent in Swedish, but it is open to everyone. The walk-in centre has been running since 2012 and receives a maximum of 45 patients daily. It is open on weekdays between 8.00 and 13.00. The patients first meet a nurse and are sometimes, if needed referred to a doctor at the healthcare centre. Interpreters in Arabic and Somali are available during the office hours of the walk-in centre, and interpreting services in other languages can be ordered on demand from an interpreter agency. A large majority of Swedish healthcare centres do not have a walk-in centre. Instead, a nurse makes an assessment of whether the patient should visit the healthcare centre over the phone, advising the caller about self-care or referring the caller to another healthcare provider.

### Patients

The following inclusion criteria were used: Swedish-born patients with experience of visiting the walk-in centre on at least one occasion. The sample was purposeful (Patton, 2015) and included 12 participants (11 female and 1 male) aged between 25 and 86 years (median age of 70). Ten participants sought care on their own behalf and two participants sought care for a sick child. In these cases, their children were present during the interview. All participants had visited the walk-in centre on several occasions, although they did not know how many times. All those who were asked gave their consent to participate with the exception of two people who said they would come to the interview after their visit, but they never showed up.

### Data collection

The head of the healthcare centre gave written permission to conduct the study. An interview guide was developed and discussed by the research team (RW, KH, EH). A semi-structured interview guide was considered useful as it gave the participants the opportunity to express their own experiences within a given framework (Patton, 2015). The guide was pilot tested on two patients and no changes were made to the interview guide, so these pilot-tested data were analysed and included in the study.

The first author participated in a workplace meeting and informed the healthcare staff about the study. The interviews were conducted in a secluded conference room at the walk-in centre. As patients mostly use the walk-in centre on Mondays, the interviews were conducted on Mondays. Patients were asked by the first author if they were willing to participate while they were in the waiting room. If they were willing to participate, they were asked to come to the conference room when their visit was finished or immediately if they had a long wait. If their queue number was called during the time of the interview, they immediately received help after the interview ended. This became relevant only in one case.

The interviews, conducted between November 2018 and January 2019, were led by a nurse and researcher in nursing (RW). Before the interviews, the patients were told about the aim of the study and were given the opportunity to ask questions about the study. Participants were also provided with a participant information sheet and were given time to read this. The idea was to partly inform the patient and partly to create a safe interview environment. In addition, written informed consent was obtained. The interviews began with one question: Can you describe your experience of being a patient at the walk-in centre? The introductory question was followed by questions about how the treatment was perceived, why they had sought care, and what expectations they had for the care that was offered at the walk-in centre. To deepen and clarify the patient’s answers (Patton, 2015), probing questions such as ‘Can you tell me more?’ or follow-up questions were asked. The interviews lasted between 10 and 50 min (median: 25 min) and were digitally recorded. After the interviews, there was time for reflection if the participant desired. In case of any unexpected reactions, there was also a readiness for further conversations with the walk-in centre’s regular staff, which was not needed. A professional secretary transcribed the interviews verbatim, inspired by a transcription guide (McLellan, Macqueen, & Neidig, 2003).

### Data analysis

The data were analysed using content analysis as described by Patton (2015). Content analysis focuses on the characteristics of language as communication, particularly the content or contextual meaning of the text. The analysis is a reasonable choice when phenomena are to be described when there is no or limited theory and literature (Hsieh & Shannon, 2005). The analysis was conducted by two researchers (RW, EH). Initially, all transcripts were read several times in order to obtain a sense of the whole. Next, the transcripts were read word by word to find and highlight words in the text that captured key thoughts or concepts related to the study’s aim. Codes were then determined and placed close to the original text. The analysis of how to categorise the different codes based on how they were related and linked continued until consensus between the researchers was reached. The subcategories were developed when similar codes were merged and finally the categories were developed. The last author (KH) read the raw data to validate the content of the categories (Patton, 2015). The results are presented with categories and subcategories supported by quotations from the participants (Patton, 2015).

### Ethical considerations

The study was approved by the Regional Ethical Review Board in Linköping, Sweden (Dnr 2017/223-31) and was performed in accordance with the Declaration of Helsinki (World Medical Association Declaration of Helsinki 2013).

### Results

Three categories were uncovered from the data: (1) several reasons for seeking care at the walk-in centre – three subcategories: referred from another healthcare provider, easy access, and help with minor health problems; (2) personal expectations determine the experience – three subcategories: expectations of the healthcare staff, bad experiences in the past, and a professional approach with good care; and (3) the walk-in centre can be developed – three subcategories: better premises, more personnel with possibilities for the development of skills, and better queuing system through person-centred assessments.

## Several reasons for seeking care at the walk-in centre

This category describes why the patient visited the walk-in centre. Therefore, the category refers to the common reasons patients sought help at the walk-in centre. Often, these were simple health problems such as upper respiratory tract infections and minor injuries.

### Referred from another healthcare provider

Most of the informants were patients at the healthcare centre for a long time and therefore were familiar with the walk-in centre. On several occasions, they had sought care when they felt they needed it. Others said that they were referred to the walk-in centre by ‘1177’, either by telephone or by the website 1177.se, which is a national telephone number for medical advice, staffed by registered nurses, open 24 h a day, seven days a week. The nurses who respond to a 1177 contact assess the need for care and give advice and guidance on further care.

“I had been ill for about three weeks and then I called 1177. Then the nurse suggested that I should go to the walk-in centre. That was not my intention, because I just wanted good advice.” (P3)

### Easy accessibility

Good access to care is presented by the majority as a reason to seek care at the walk-in centre. Knowledge that they can contact caregivers on precisely the day they want creates a feeling of security, even if there is a long waiting time. For some patients with hearing loss or other impairments that cause problems with using a phone, it is easier to seek care directly at the walk-in centre than to speak over the phone. Others believed that scheduled appointments at the healthcare centre are difficult to obtain, which is why the walk-in centre is used.

“I think it’s really hard to use the phone. You have to listen to different instructions, and you have to press here and you have to press there […] when you don’t hear [well] it is very difficult,” (P9)

### Getting help with minor health problems

The patients who seek the walk-in centre consider that they have good knowledge of the possibilities and limitations of the care they can receive. They do not seek treatment for acute symptoms and many say that they will not seek care at the walk-in centre if they think their problems require lengthy assessment. In other words, they use the walk-in centre for minor health problems. There are many reasons for visiting the walk-in centre, such as prolonged colds, symptoms of the flu, wax plugs, and muscle aches.

“This was a prolonged cold for me for almost a month and for my daughter for almost two weeks. And then, when I read, it says that you should […] if it has been more than 10 days, then you should go and check. So that’s why […].” (P1)

## Personal expectations determine the experience

This category describes perceptions of the care they have received through their visits to the walk-in centre. All informants had previously visited the walk-in centre – some more often, some on fewer occasions. Therefore, experiences emerged that were not based solely on one care episode.

### Expectations of the healthcare staff

The patients had many expectations of the healthcare staff. Ultimately, the patients expect healthcare staff to be competent and have a respectful attitude by listening carefully and offering adequate help. As nurses receive patients first, it is important that they have the ability to decide what they can manage themselves and when they need to consult a doctor.

“I think they [health care staff] should be responsive […] open to what I’m saying and listen carefully. Not just think that I’m here unnecessarily […] which perhaps many are.” (P11)

Several patients stated that it is important that their nurse is friendly and skilled, and some believed that this is extra important when children need care. In addition, the patients desired that the nurses spend enough time with them, allowing them to ask follow-up questions, to create a better assessment.

### Bad experiences in the past

Although most of the patients were pleased with the walk-in centre, some expressed negative experiences. For example, some participants said they had problems understanding the healthcare providers who spoke limited Swedish or that they felt they were not listened to, which was thought to be due to the stress the healthcare providers were experiencing. Above all, it was the long waiting times that many described as a disadvantage. Even if they could handle the waiting time themselves, they thought other patients might not be able to cope. For example, the elderly, the mentally ill, and people with jobs may have a hard time waiting. Some recalled parents of sick children waiting a long time for their turn.

“It [waiting with a child] was a disaster! When I came here in the morning […], [the waiting area was] just packed with patients everywhere. I was standing in the lobby […] hanging around. […] It was a really long wait.” (P4)

One other aspect raised by several informants is a fear of being infected since patients carrying airborne infection are waiting in the same waiting room. This is perceived as stressful as they do not want to complicate their health problems. One patient mentioned that there was no other option than seeking care at the walk-in centre as it is difficult to schedule a time that will work at the traditional healthcare centre.

### A professional approach with good care

Most of the informants said that the care they received was professional, offered rapid physical examinations on site, and was highly accessible. The professional approach seems to include a nurse or a doctor who is a good listener, friendly, experienced, and skilled. The latter may mean that the care provider recognises his or her limitations and can refer the patient for other assessments or treatment if necessary.

“I am always treated kindly; they are so accommodating and competent.” (P6)

Some also emphasise the importance of having this type of care for those who do not speak Swedish or for other reasons have difficulties getting in touch with the care providers via telephone. This means that everyone can be assessed even if they are not fluent in Swedish.

“It’s difficult when you can’t speak Swedish and you might not be technically skilled or […] understand. So, in that way it’s good. But for me who would like to book a time, it is a little disturbing to have to wait here for so long […] but it’s the rules of the game.” (P8)

## The walk-in centre can be improved

The participants suggested several ways to improve the walk-in centre experience. In part, these suggestions are about expanding the walk-in centre so that more care can be provided with shorter waiting times. The queuing system, the premises, and how care can be organised where people of many different origins gather are a few other factors that are described below.

### Better premises

The informants mentioned several ways to improve the premises. The proposals were partly about providing children with books, toys, and games. However, they also suggested ways to improve the waiting experience for adults, such as supplying enough chairs so everyone can sit, providing a TV in the waiting room, and playing background music.

If patients were to be triaged directly on arrival, infectious patients could be secluded in a special waiting room. However, some also suggested different waiting rooms or departments for Swedes and immigrants. In addition, many expressed irritations with immigrants bringing their relatives to the waiting room and not controlling the behaviour of their children.

“There was one person who was sick and there were six people [relatives] who came along. […] A lot of people who are not sick moving here and there. I’m not a racist in any way, but they should conform to our [Swedish] customs as well.” (P2)

### More personnel and possibilities for the development of skills

Some participants believed that the walk-in centre should be open all day and not serve a limited number of patients. However, this would require more staff. Having several nurses and doctors connected to the walk-in centre would mean more patients could get help and therefore shorten queuing time

“Getting more staff would be great. Partly to reduce their workload […] but also for patients, of course.” (P7)

To offer as good and efficient care as possible, some suggested that the staff should receive tutorials at the end of the work session, so they could learn from each other to improve the way of working.

“The staff surely meet lots of people with different medical conditions; it would be good if they learned from each other’s experiences.” (P5)

### Better queuing system through person-centred assessments

All participants had to use the queue system at the walk-in centre and they had several initiatives and suggestions for how to improve the queue system. One of the suggestions is to introduce some form of triage to determine whether someone needs to be attended to more urgently. Several participants also discussed issues related to children. Children in the waiting room can disturb or cause concern for other care seekers, so one of the suggestions was to let the children see care providers as soon as possible.

“There is nothing to do for the children. No children’s books, no children’s corner. Then three hours [of waiting] will be a very long time.” (P1)

Having a display that shows queue numbers outside the walk-in centre would allow patients to get out and breathe fresh air while waiting their turn. Similarly, care providers could notify waiting patients when they are on break so the patients can step outside during this time. Another suggestion is that patients receive a text message when it is their turn, a strategy that could reduce the time in the waiting room and therefore reduce the risk of being infected by others.

“It is pointless to sit and wait; [it is] better to be able to go out and come back when it is my turn.” (P10).

## Discussion

This study evaluates a walk-in centre at a healthcare centre in an immigrant-dense area from the perspective of Swedish-born patients. This study is unique as it evaluates a walk-in centre at a healthcare centre in the context of immigrant care seekers from the perspective of Swedish-born patients. These Swedish-born patients offered suggestions about how to organise PHC that is primarily not designed for them. As the literature review did not reveal any previous studies on this topic, only partial comparisons to previous studies will be possible.

The main finding of this study is that the walk-in centre is accepted by the Swedish-born patients as almost everyone could see the benefits of the system. Above all, the benefits relate to good accessibility and that the system is understood as a way to make care equal and possible for everyone, such as for people who are not fluent in Swedish or who have other reasons for not being able to seek care by phone. It seems that the informants themselves would have preferred a different system, such as ‘a classic health care centre’; however, the system has legitimacy because everyone can use it. Amongst the top 10 research priorities in PHC, O’Neill *et al.* (2018) emphasise just how PHC best can address the social determinants of health and promote health equity.

However, the participants were not entirely positive. Although the walk-in centre is appreciated for simpler conditions due to its good accessibility, many suggestions address how it can be developed. The long waiting times and overcrowded waiting rooms are described as making it difficult for employed persons and families with children. Anderson, Camacho, and Balkrishnan (2007) found that longer wait times were associated with lower patient satisfaction. However, the time spent with a physician was a stronger predictor of patient satisfaction than the time spent in the waiting room.

The participants also feared being infected by other patients in the waiting room. It is well known that waiting rooms can be problematic based on the risk of infection spread. For example, Beggs *et al.* (2010) found that waiting areas of healthcare facilities present a particular challenge, since large numbers of patients, some of whom may have underlying conditions that predispose them to infection, can be exposed to an individual who may be shedding potentially pathogenic microorganisms. As suggested by some of our informants, an outdoor display that shows the queue number or a system connected to the mobile phone that tells waiting patients when it is their turn could reduce infection risk. The technique already exists and perhaps this will receive further support considering the recent outbreak of COVID-19.

Most informants revealed that they received professional care from a nurse or a doctor who was a good listener, friendly, experienced, and skilled. At the same time, the opposite was also described. Some informants revealed that their caregivers did not listen to them and were perceived to be stressed. In summary, a more pessimistic picture appears in this evaluation made by the Swedish-born patients compared to a previous study where the staff at a walk-in centre evaluated the system (Wärdig *et al.*, 2019). This discrepancy between studies could be because the walk-in system was not primarily designed for Swedish-born patients or that healthcare personnel are more positive about the system that they themselves represent.

The results of this study reveal a clash of cultures. For example, some Swedish-born informants felt that the immigrants ignore Swedish norms by bringing family members with them when they come to the walk-in centre. Some informants also felt that the children of immigrants often behave in a manner unsuitable for a healthcare facility. The healthcare system has increasingly been confronted by patients from many cultures (Flores, 2000). Cultural clashes can be understood as conflicts of philosophies, styles, values, and missions. It is not about determining what is right or wrong; rather, it is about when ignorance and prejudice from one of the cultures come to the fore in lieu of seeking understanding (Nguyen & Kleiner, 2003). It is somewhat surprising that several informants proposed separate waiting rooms for Swedish-born and immigrant patients. This suggestion is undesirable, will not be realised, and would violate the Health and Medical Services Act (Ministry of Health and Social Affairs, 2017). According to this act, health care must be given with respect for the equal value of all people and for the dignity of the individual.

The researcher (RW) made the initial approach in recruiting potential informants while they waited for their turn. We can see that there are risks associated with informants feeling obligated to participate in this procedure. At the same time, the informants received both oral and written information that participation was voluntary, and signed informed consent before participating. There were difficulties finding informants for the study. Most of the care applicants had a foreign background, so the interviewer (RW) visited the healthcare centre on several occasions without finding anyone who met the inclusion criteria. This does not reflect the population, although there are many immigrants in the area (Statistics Sweden, 2016). Because of this, the number of informants can be regarded as somewhat low in relation to the method employed (Patton, 2015). At the same time, the material was perceived as sufficient for analysing the data, as the categories emerged clearly. The analysis showed that the same content was repeated, providing a homogenous picture, which enhances its trustworthiness (Patton, 2015). However, the selection of informants also raises other questions as the sample was relatively homogeneous. What does it mean that all respondents, except one, were women? How does the relatively high age of the informants affect the results? Where are the employed and where are the men? If younger people and employed people do not use the walk-in centre, perhaps, the care must be arranged in a different way. It might be unreasonable for them to wait several hours for their turn. Perhaps, these groups need to be offered other solutions to increase accessibility. Computer-based, mobile solutions, or extended opening hours could possibly help these groups of potential patients’ access health care more easily and equitably. By increasing the use of digital technologies to inform, support, and build capacity, the healthcare providers could be empowered to improve the quality of care in PHC. In addition, these strategies could encourage patients to take an active role in their health and well-being (WHO, 2018). However, the challenge remains to meet the needs of mainly low-educated people with low social positions, whether Swedish-born or immigrants.

One strength of qualitative studies is their ability to gain a deeper understanding of the phenomenon studied, but the main limitation is that the results cannot be generalised (Patton 2015). Could a larger structured study based on this qualitative study produce other results? To address this question, we plan, in the series of studies evaluating the walk-in centre, to implement a survey that will more clearly identify who and why people seek care at walk-in centres. Nurses or interpreters will ensure that the patient understands the questions and can answer them regardless of origin.

A strength of this study is that the interviewer had no previous connection with the patients or the staff at the walk-in centre. As the interviewer did not know in advance what problems the patients may have, there was a readiness for further support through regular nurses at the healthcare centre, but this support was not needed or used.

## Conclusion

The Swedish-born patients appreciated some aspects of the walk-in centre. For example, they found that the walk-in centre was accessible and provided professional care for minor health problems. Past experience may determine how the patients viewed the walk-in centre and the quality of care provided. Many suggestions were made to improve the service. The proposals included reducing the spread of infection by providing the possibility to wait outdoors and recommending that patients not bring their relatives to the walk-in centre if possible. In addition, some suggested that some form of triage should be used that prioritises children as well as more difficult cases. However, because the informant group was very homogeneous (i.e., mainly retired women), many questions about accessibility need to be addressed.
